# Role of Nanotechnology in Ischemic Stroke: Advancements in Targeted Therapies and Diagnostics for Enhanced Clinical Outcomes

**DOI:** 10.3390/jfb16010008

**Published:** 2025-01-01

**Authors:** Virendra Kumar Yadav, Rachna Gupta, Abdullah A. Assiri, Jalal Uddin, Azfar A. Ishaqui, Pankaj Kumar, Khalid M. Orayj, Shazia Tahira, Ashish Patel, Nisha Choudhary

**Affiliations:** 1 Marwadi University Research Center, Department of Microbiology, Faculty of Sciences, Marwadi University, Rajkot 360003, Gujarat, India; 2 Department of Pharmaceutics, National Institute of Pharmaceutical Education and Research (NIPER), Ahmedabad 382021, Gujarat, India; rachna100008@gmail.com; 3 Department of Clinical Pharmacy, College of Pharmacy, King Khalid University, Abha 61441, Saudi Arabia; aalabdullah@kku.edu.sa (A.A.A.); azfar.hd@hotmail.com (A.A.I.); korayg@kku.edu.sa (K.M.O.); 4 Department of Pharmaceutical Chemistry, College of Pharmacy, King Khalid University, Abha 61441, Saudi Arabia; jalaluddinamin@gmail.com; 5 Department of Environmental Science, Parul Institute of Applied Sciences, Parul University, Vadodara 391760, Gujarat, India; pankajb434@yahoo.com; 6 Institute of Professional Psychology, Bahria University Karachi Campus, Karachi 75260, Pakistan; shaziatahira@gmail.com; 7 Department of Psychiatry, Jinnah Postgraduate Medical Centre, Karachi 75510, Pakistan; 8 Department of Life Sciences, Hemchandracharya North Gujarat University, Patan 384265, Gujarat, India; uni.ashish@gmail.com; 9 Department of Lifesciences, Parul Institute of Applied Sciences, Parul University, Vadodara 391760, Gujarat, India

**Keywords:** nanomedicine, drug delivery, nanomaterials, stroke, blood–brain barrier

## Abstract

Each year, the number of cases of strokes and deaths due to this is increasing around the world. This could be due to work stress, lifestyles, unhealthy food habits, and several other reasons. Currently, there are several traditional methods like thrombolysis and mechanical thrombectomy for managing strokes. The current approach has several limitations, like delayed diagnosis, limited therapeutic delivery, and risks of secondary injuries. So, there is a need for some effective and reliable methods for the management of strokes, which could help in early diagnosis followed by the treatment of strokes. Nanotechnology has played an immense role in managing strokes, and recently, it has emerged as a transformative solution offering innovative diagnostic tools and therapeutic strategies. Nanoparticles (NPs) belonging to several classes, including metallic (metallic and metal oxide), organic (lipids, liposome), and carbon, can cross the blood–brain barrier and may exhibit immense potential for managing various strokes. Moreover, these NPs have exhibited promise in improving imaging specificity and therapeutic delivery by precise drug delivery and real-time monitoring of treatment efficacy. Nanomaterials like cerium oxide (CeO_2_) and liposome-encapsulated agents have neuroprotective properties that reduce oxidative stress and promote neuroregeneration. In the present article, the authors have emphasized the significant advancements in the nanomedicine management of stroke, including NPs-based drug delivery systems, neuroprotective and neuroregenerative therapies, and multimodal imaging advancements.

## 1. Introduction

Strokes are one of the major leading causes of death and disability worldwide, which presents significant challenges in both prevention and treatment. Without a doubt, continuous advances have been made in medical research and healthcare; still, the complexity of stroke pathophysiology and the variability in patient response to treatment continue to pose substantial hurdles [1]. The trademark characterization of stroke is a sudden interruption of blood flow to the brain, which remains a noteworthy worldwide health challenge with profound implications for morbidity and mortality [2]. Broadly, strokes can be classified into ischemic strokes (IS) and hemorrhagic strokes. An ischemic stroke is caused by the blockage of blood vessels supplying the brain, while a hemorrhagic stroke occurs due to the rupture of blood vessels within the brain. Both types of strokes can lead to severe neurological deficits and long-term disability if not promptly diagnosed and treated [3].

Among the currently available methods for diagnosing and treating strokes, Endovascular Treatment (EVT), intravenous thrombolysis, nanotechnology [4], and carotid and intracranial atherosclerotic disease management are widely used [5,6]. A prevalent feature of current stroke treatment refers to time-sensitive treatments that focus on restoring blood flow to the affected brain region. Thrombolytic agents like tissue plasminogen activator (tPA) or mechanical thrombectomy (MT) are often needed to eliminate clots in IS. In the case of hemorrhagic strokes, there is a need for surgical interventions to repair damaged blood vessels and reduce intracranial pressure [7,8]. All these current available techniques have drawbacks; for instance, EVT is a highly effective treatment for acute IS, but its implementation around the globe is still very limited. Some major issues are lack of access, selective undertreatment, and logistical bottlenecks in healthcare systems. Moreover, several issues are being faced for further expansion of the EVT services, like allocation of resources, training, and infrastructure development [9]. Intravenous thrombolysis is essential for acute stroke therapy, although it remains limited by a limited therapeutic window and limitations in specific patient groups. The need for rapid diagnosis and prompt therapy initiation prevents its extensive applications [10,11]. The management of carotid stenosis and intracranial atherosclerotic disease raises various hurdles due to the complexity of surgical and endovascular interventions. Treatment decisions typically depend on outdated clinical trial data, demanding customized approaches suitable to individual patient profiles [12].

Besides this, there are several emerging approaches for treating strokes, like stem cell therapy (SCT), neurorestorative approaches, and pediatric stroke treatments [2,13]. Stem cell transplantation offers a promising avenue for neuroprotection and neurorestoration, potentially replacing damaged neurons, reconstructing nerve pathways, and modulating the immune response [14]. However, challenges like tissue sourcing, delivery methods, and immune reactions must be addressed to optimize its efficacy [15]. Brain organoid culturing, animal models, and biomaterial advancements open the door to neurorestorative strategies. Although significant pathobiological and technological hurdles remain, these strategies aim to repair structural brain damage and restore function [16]. Innovative treatments, including SCT and robotic rehabilitation, are being explored for pediatric stroke. These strategies aim to enhance functional recovery and reduce long-term disability, highlighting the importance of tailored interventions for this population [17].

So, from the above, it is found that all the currently available therapeutic approaches to strokes have several limitations. Some of the common challenges with current stroke management approaches have delayed diagnosis, the risk of secondary brain injury, and the challenge of delivering therapeutic agents directly to the affected site within the brain. Symptomatic non-stenotic carotid plaques are an under-recognized stroke risk factor [18,19]. The lack of standardized definitions and imaging protocols complicates diagnosis and treatment, which requires further studies to establish effective management strategies [20]. Post-stroke rehabilitation is one of the major challenges, with persistent motor and cognitive deficits. Non-invasive neurostimulation techniques, like rTMS and tDCS, demonstrate promise in promoting recovery by modulating brain activity and increasing neuroplasticity [21]. The current stroke treatments have improved the rate of survival, but they often fall short of addressing long-term functional impairments. The complicated nature of stroke pathophysiology and patient diversity requires a comprehensive strategy incorporating conventional and innovative medicines. Continuous research and innovation are needed to address existing challenges and enhance outcomes for stroke patients. A developing strategy in stroke identification and treatment is the use of nanoparticles (NPs) and nanotechnology, which may be essential due to their small size, significant surface area to volume ratio (SVR), and ability to pass through the blood–brain barrier (BBB) [22,23].

Nanotechnology manipulates materials at the nanoscale, often less than 100 nm, to produce medically useful particles [24]. These NPs can be surface functionalized to increase their affinity and specificity for biological targets, enabling precise diagnostic and therapeutic actions with minimal adverse effects [25]. Nanotechnology is revolutionizing medicine, offering new ways to diagnose and cure disorders, including stroke [26]. Nanotechnology may improve BBB drug delivery for stroke patients, a major challenge in neurological disease treatment [27]. The integration of nanotechnology into medical applications, particularly in stroke management, holds promise for overcoming these obstacles by improving drug delivery, enhancing diagnostic accuracy, and facilitating targeted therapies [28,29].

NPs could be used for targeted drug delivery for the management of stroke, where they could be engineered for the direct delivery of drugs to the site of ischemic injury [28,30]. This will improve therapeutic results by concentrating the treatment where it is most needed. For instance, bioactive NPs have been synthesized to deliver antioxidative and anti-inflammatory agents to the brain, which reduces neuronal apoptosis and promotes recovery in IS models [31]. Moreover, these NPs could play a significant role in enhancing stem cell therapies. Nanomedicine can also improve the efficacy of SCT for stroke by directing the differentiation of neural stem cells into functional neurons rather than astrocytes, thereby improving structural and functional recovery [32]. This is achieved by delivering NPs that silence specific long noncoding RNAs, facilitating neuronal differentiation and allowing for in vivo tracking of stem cells [33].

Various organic nanomaterials are potential candidates for diagnosis and therapeutic applications in stroke therapy. For instance, nanobodies, a novel group of single-domain antibodies, provide enhanced tissue penetration and have a superior ability to cross the BBB compared to conventional antibodies [30]. These nanobodies can influence neuroinflammatory processes, which are essential in the pathophysiology of stroke, thereby serving a dual function in both diagnosis and treatment [30]. Additionally, NPs may significantly contribute to genetic and molecular therapies. NPs have been utilized to transport anti-miR oligonucleotides that target specific microRNAs associated with stroke pathology. This method has shown potential in decreasing infarct size and enhancing outcomes in preclinical models. The utilization of NPs for the delivery of genetic material or small chemicals is an innovative method for influencing the molecular pathways associated with stroke [34].

The present review explores the foundational aspects of nanotechnology and its specific applications in stroke management. The authors have also reviewed the potential of nanomedicine to revolutionize stroke therapy. Further, emphasis was given to the importance of nanotechnology in revolutionizing the treatment and diagnosis of strokes. Such a review will help to address current stroke care limitations, improve patient outcomes, and pave the way for more personalized and effective therapeutic strategies in neurology.

## 2. Blood–Brain Barrier (BBB)

The BBB is mainly made up of non-wall-less endothelium characterized by tight junctions, forming a cell barrier that is almost impossible to penetrate [35]. Brain capillary endothelium (BMECs) has obvious structural features compared to other organs’ capillary [36]. Firstly, there is a lack of pores in the capillary, and the overlapping coverage and tight junctions between the cells decrease the passage of the drug by a larger amount. Secondly, the endothelial cells are surrounded by a continuous layer of basement membrane, and thirdly, there are several perivascular feet of astrocytes outside the basement membrane. All of these components form the capillary membrane of the brain, which forms the protective BBB of the brain tissue. Currently, the major pathways through BBB include passive diffusion, adsorption-mediated transport, receptor-mediated transport, carrier-mediated transport, and cell-mediated transport. Table 1 shows the key points among the different pathways through BBB.

## 3. Pathophysiology of Stroke and Its Types

Stroke is a significant central nervous system (CNS)-related disorder characterized by the sudden disruption of blood flow to the brain, causing damage to brain tissue [37]. One of the major hurdles in stroke is the restricted regenerative capacity of neurons in the affected brain region [38,39]. The brain’s capability to repair and regenerate damaged neurons is inherently complicated, and this often leads to long-term disability in people who have experienced a stroke [40,41]. There is the development of ischemia, angiogenesis, and nerve growth, which are severely restricted, resulting in an ischemia cavity in the brain [42]. From the various pieces of literature, it has been revealed that about 87% of strokes result from ischemia [43,44], and the remaining 13% are caused by hemorrhage [45]. In the deficiency of blood in the brain, the neurons and neural circuits become damaged, leading to IS and behavioral disorders. The vascular endothelial growth factor concentration is directly related to BBB disruption and brain edema [42].

Therefore, one of the major challenges in treating stroke is the difficulty in forming intracranial blood vessels [46]. In current efforts to address stroke with tissue repair technologies, the main focus sites include the adjacent region of the stroke cavity, the area encompassing the infarction, and regions displaying robust nerve and vascular plasticity after a stroke [47]. Currently, ISs are treated by clearing the thrombus (ThB) with mechanical approaches and intravenous injection (IVI) tissue-type plasminogen activators [48].

### 3.1. Types of Strokes

**Figure 1:**
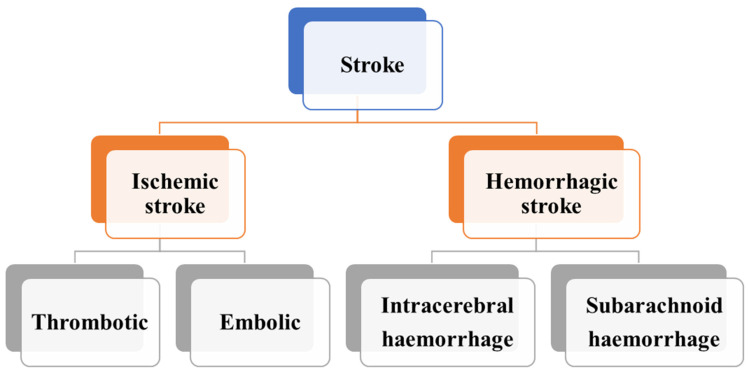
Different subtypes of strokes.

Strokes, or cerebrovascular accidents (CVAs), are categorized into two primary types: ischemic and hemorrhagic [41,49,50], which are mentioned briefly below and shown in Figure 1.

#### 3.1.1. Ischemic Strokes

Ischemic strokes constitute about 87% of all strokes and occur when a blood vessel supplying the brain is obstructed [6,51]. This obstruction can be due to a thrombus (a blood clot forming within the blood vessel) or an embolus (a clot or debris traveling from another part of the body and lodging in the brain’s vasculature). The resulting reduction in blood flow leads to a deficiency in oxygen and nutrients to the affected brain tissue, causing cell death. Common causes include atherosclerosis, atrial fibrillation, and carotid artery disease [52].

#### 3.1.2. Hemorrhagic Strokes

Hemorrhagic strokes occur when a blood vessel in the brain ruptures, leading to bleeding within or around the brain [53,54]. These strokes are further classified into intracerebral hemorrhage (bleeding within the brain tissue) and subarachnoid hemorrhage (bleeding in the space between the brain and the surrounding membrane). Hypertension, aneurysms, arteriovenous malformations, and head trauma are typical causes. The bleeding increases intracranial pressure, damaging brain cells and disrupting normal brain function [55].

### 3.2. Mechanisms of Brain Injury and Neurological Deficits

Regardless of the type, the fundamental consequence of a stroke is the disruption of normal blood flow to parts of the brain, resulting in tissue damage and loss of function in the affected areas.

#### 3.2.1. Ischemic Strokes

The blockage of blood flow in ischemic strokes results in a cascade of events leading to brain injury [56,57,58]. The immediate lack of oxygen (hypoxia) and glucose triggers a series of metabolic disturbances. Neurons begin to die within minutes due to energy failure, releasing excitatory neurotransmitters like glutamate. This causes excitotoxicity, where excessive calcium influx into neurons leads to further cellular damage and death. Additionally, the affected area, known as the ischemic core, is surrounded by the ischemic penumbra, where cells are at risk but still salvageable with timely intervention [59].

#### 3.2.2. Hemorrhagic Strokes

The main injuring mechanism in hemorrhagic strokes is the direct damage caused by the hemorrhage. Blood extravasation results in raised intracranial pressure and mechanical injury to brain tissues [49,60]. The breakdown of the blood products may cause inflammation and cytotoxicity, exacerbating neuronal damage. Furthermore, the pressure from the collecting blood could end up in herniation, when parts of the brain move into neighboring compartments, so further affecting neurological processes [61].

#### 3.2.3. Neurological Deficits

**Figure 2:**
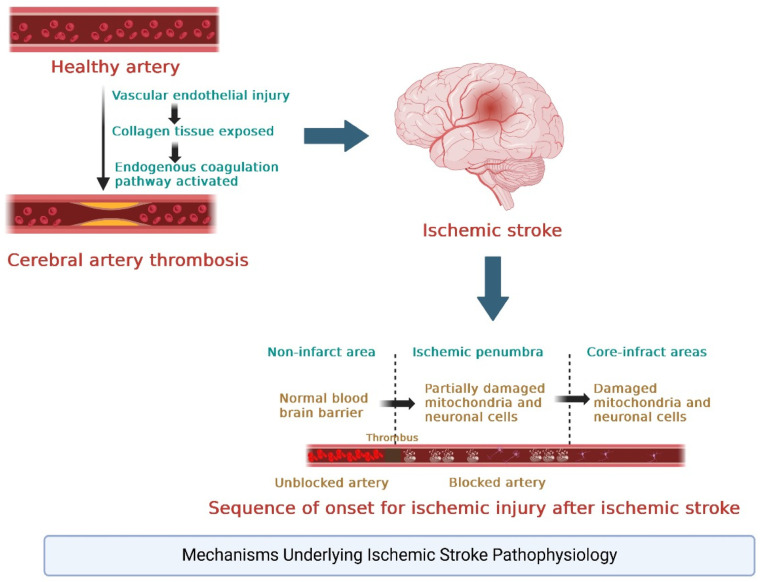
Pathophysiology of ischemic stroke.

A stroke’s clinical manifestations depend on the affected brain region. Common deficits include hemiparesis or hemiplegia (weakness or paralysis on one side of the body), aphasia (language difficulties), dysarthria (difficulty speaking), vision disturbances, and cognitive impairments. The severity and type of deficits provide insight into the stroke’s location and extent of damage. For instance, a stroke in the left hemisphere may result in language deficits (Broca’s or Wernicke’s aphasia), while a stroke in the right hemisphere may cause spatial neglect [62,63]. Figure 2 shows the pathophysiology of ischemic stroke.

## 4. Current Approaches to Stroke Diagnosis and Treatment

Significant advances in stroke diagnosis and treatment have been achieved through nuclear medicine and imaging technologies, particularly with the development of mobile stroke units, Magnetic Resonance Imaging (MRI)-based techniques for four-dimensional (4D) flow imaging, and the identification of pre-hospital acute stroke biomarkers. Incorporating nuclear medicine (Positron Emission Tomography) into multimodal imaging devices (Computed Tomography and MRI), like PET-CT and PET-MRI, enables the generation of more comprehensive brain images by combining functional and anatomical information. Such developments have improved clinical outcomes following stroke and shortened treatment delays. Moreover, combining innovative PET with Glycoprotein 1 and radionuclide angiography has boosted the specificity and sensitivity of nuclear machines in detecting brain stroke [64].

### 4.1. Diagnostic Techniques

Several diagnostic techniques are available for quick diagnosis and triage of stroke scales. These established clinical ratings allow patients suspected of having acute IS to be quickly diagnosed. Aiming to reduce brain damage and enhance patient outcomes, prompt and suitable stroke treatment depends on an accurate diagnosis. Important methods of diagnosis include a CT scan, MRI, and other techniques.

#### 4.1.1. Computed Tomography (CT) Scan

As a CT scan can be obtained quickly and is widely available, it is usually the first imaging method employed in emergencies. By detecting brain bleeding, it assists in differentiating between ischemic and hemorrhagic strokes. Endovascular therapy (EVT) patients are frequently selected for treatment using computed tomography angiography (CTA) and computed tomography perfusion (CTP) [65]. Time consumption for sequential imaging is the main disadvantage of this approach, although time reduction is being made easier by the development of automated software. Compared to CTA and CTP, non-contrast CT is cost-effective, fast, and excellent for detecting acute hemorrhage. It can identify a massive stroke but is notably insensitive when identifying small strokes [66].

#### 4.1.2. MRI

The most sensitive imaging technique is especially helpful in diagnosing IS and yields more detailed images of brain tissue than CT scans [67]. As soon as symptoms appear, diffusion MRI, also known as diffusion-weighted imaging (DWI), is highly sensitive in detecting early ischemic changes. DWI shows the diffusion of protons throughout the tissue with a low apparent diffusion coefficient that appears to be those with restricted or delayed proton transport [68].

The perfusion fraction associated with cerebral blood volume can be obtained using DWI. The sizes of the core and penumbra can be assessed via non-contrast CT (NCCT), CTA, and CTP [69,70]. T2-weighted MRI and susceptibility-weighted imaging (SWI) can effectively identify stroke and hemorrhage. The cerebral vasculature is illustrated in SWI by the phase difference resulting from the magnetic susceptibility between oxygenated and deoxygenated blood [69]. Magnetic resonance angiography (MRA) is an alternative to CTA for assessing brain perfusion. The brain’s at-risk areas, known as the penumbra, can be delineated, and MRA and Magnetic Resonance Perfusion (MRP) imaging assess blood flow [71].

#### 4.1.3. Other Techniques

Although commonly employed for diagnosing acute ischemic stroke, CT and MRI are not always accessible in medical facilities. Consequently, numerous advanced portable devices with elevated sensitivity and specificity are being developed for stroke diagnosis. Alternative techniques for imaging, such as microwave tomography, Doppler ultrasound, and volumetric impedance phase-shift spectroscopy, are employed in developing diagnostic instruments [72,73]. The microwave-based device is a compatible stretcher with 100% sensitivity and 75% specificity in detecting brain hemorrhage. This device utilizes mathematical algorithms to process signals alongside low-power, non-ionizing microwave radiation [74]. Cerebral blood flow velocity is measured using transcranial Doppler ultrasonography. It has been shown that individuals with large vessel occlusions (LVOs) can be distinguished from healthy controls with 100% sensitivity and specificity (86%) utilizing transcranial Doppler ultrasound. When diagnosing LVOs in individuals suspected of having a stroke, it had 91% sensitivity and 85% specificity [75]. A volumetric impedance phase shift spectroscopy with high sensitivity (93%) and specificity (92%) can distinguish between minor and severe LVO-caused strokes. The machine operates by sending out low-intensity radio waves on each side from the back of the head. Subsequently, the receiver positioned anteriorly in the brain receives these waves. Different brain disorders have distinct signatures produced by modifying radio waves as they pass through different tissues, depending on the kind of tissue and the fluid characteristics [75].

### 4.2. Pharmacological and Mechanical Treatments

The major objective for the treatment of strokes is restoring blood supply to the brain, stopping more damage, and managing complications. The use of thrombolytic therapy, antiplatelet drugs, and endovascular MT to mechanically remove the occlusive clot are approved therapeutic methods for the treatment of acute ischemic stroke [6].

#### 4.2.1. Ischemic Stroke Treatments

(a) Thrombolytic therapy

When a vessel is wounded, the body forms a loose platelet plug. This is followed by the activation of the coagulation cascade, which creates a fibrin mesh that reinforces the clot and permits the vessel to be repaired. Clot disintegration happens spontaneously as plasminogen is converted to plasmin, a potent fibrinolytic serine protease. At the site of damage, endothelial cells produce plasmin, which is slowly released after the plasminogen is cleaved by the tissue plasminogen activator (tPA) [76]. The most successful treatment for IS is the clot-busting drug tissue plasminogen activator (tPA). Recombinant tPA can break the clot and restore blood flow when administered within a specific time window (typically, 4.5 h of symptom onset), greatly improving outcomes [77]. However, the risk of bleeding and stringent qualifying requirements restrict its use. Although platelet-rich clots may be more resilient to thrombolysis, thrombi rich in red blood cells appear more susceptible to rtPA [78].

(b) Antiplatelet therapy

Aspirin is a regularly prescribed antiplatelet drug that stops new clot development following an IS. This drug works by permanently blocking the enzyme cyclo-oxygenase (COX), which in turn prevents platelets from aggregating by preventing the production of the procoagulant thromboxane A2 (TXA2) [79]. There was no discernible increase in hemorrhagic stroke or transformation during the 48 h following stroke. However, aspirin therapy significantly decreased the overall risk of early recurrent stroke (7/1000) and mortality (4/1000). Clopidogrel is another significant antiplatelet drug that belongs to the second generation of thienopyridines. It is a prodrug that the hepatic cytochrome P450 system metabolizes to its active form. The active metabolite inhibits the P2Y12 class of adenosine diphosphate (ADP) receptors on platelet surfaces in an irreversible manner. This inhibits ADP-mediated activation of the glycoprotein IIb/IIIa complex downstream, reducing platelet aggregation [80]. Thus, both drugs are effective in treating acute ischemic stroke and are approved by the Food and Drug Administration (FDA).

(c) Mechanical thrombectomy

In an MT, an endovascular procedure, a medical device is inserted into the vasculature through the groin, threaded through the heart to reach the cerebral vessel blocked by the blood clot, and then mechanically removed to restore normal blood flow to the brain. Depending on the patient’s eligibility, it is often carried out 6 to 24 h after the stroke begins. The Merci retriever was the first MT tool approved by the FDA to treat acute ischemic stroke patients [81]. A few years later, the penumbra system became the second thrombectomy device approved by the FDA; it demonstrated safe and effective recanalization in acute ischemic stroke patients treated within eight hours of onset of symptoms [82]. One major benefit of rtPA is its accessibility in stroke centers without MT services or 24-h care.

#### 4.2.2. Hemorrhagic Stroke Treatments

A hemorrhagic stroke occurs when a cerebral blood vessel suddenly bursts and spills blood into the nearby tissue. It hampers the brain’s regular blood flow, depriving the cells of oxygen and blood. The brain’s pressure rises due to the blood leak, crushing the cells and tissue [83]. The stroke’s location within the brain (intracerebral) or on the surface between the brain and skull (subarachnoid) determines the course of treatment [84].

(a) Surgical interventions

There are various surgical procedures for the treatment of hemorrhagic stroke, including stereotactic aspiration, endoscopic aspiration, decompressive craniectomy, and catheter aspiration. Surgery like hematoma evacuation or craniotomy (removal of a portion of the skull) may be required in cases of severe bleeding or high intracranial pressure [85]. Endovascular techniques like coiling or clipping repair aneurysms or arteriovenous malformations. Stereotactic aspiration is one of the minimally invasive procedures that is being tested. In patients with spontaneous putaminal bleeding, whose eyelids will open in response to powerful stimuli, Hattori et al. demonstrated in randomized research the usefulness of stereotactic evacuation [86].

(b) Drugs

The control of blood pressure is very important for hemorrhagic stroke patients to prevent bleeding. Using beta-blockers (labetalol, esmolol), an ACE inhibitor (enalapril), a calcium channel blocker (nicardipine), or hydralazine, blood pressure should be gradually brought down to 150/90 mmHg [87]. Inflammation, oxidative stress (OS), and toxicity of erythrocyte lysates and thrombin are the reasons for stroke. Drugs like pioglitazone, misoprostol, celecoxib, edaravone, flavanoid, nicotinamide, and mononucleotide reduce inflammatory damage and OS [88]. Furthermore, if the patient is taking blood-thinning medicine, drugs (such as vitamin K or prothrombin complex concentrates) may be given to offset the effects of anticoagulants.

#### 4.2.3. Supportive and Rehabilitative Care

Proper nursing, rehabilitative, and medical care are crucial for the management of hemorrhagic stroke [89]. Common problems include aspiration, difficulty swallowing, heart rhythms, stress-induced cardiomyopathy, heart failure, acute kidney damage, infection in the gastrointestinal tract, urinary tract infections, and many more. It could be necessary to have a percutaneous endoscopic gastrostomy to stop aspiration. It is advised to screen for myocardial ischemia in hemorrhagic stroke patients using an ECG and cardiac enzyme tests. While elastic stockings may not be very helpful, intermittent pneumatic compression lowers the risk of deep vein thrombosis. Multidisciplinary rehabilitation is suggested to reduce the disability. Monitoring blood sugar levels and taking precautions to avoid hyperglycemia and hypoglycemia is important. Regardless of stroke type, comprehensive care involves supportive measures and rehabilitation [90].

## 5. Nanotechnological Advances in Stroke Diagnosis

**Figure 3:**
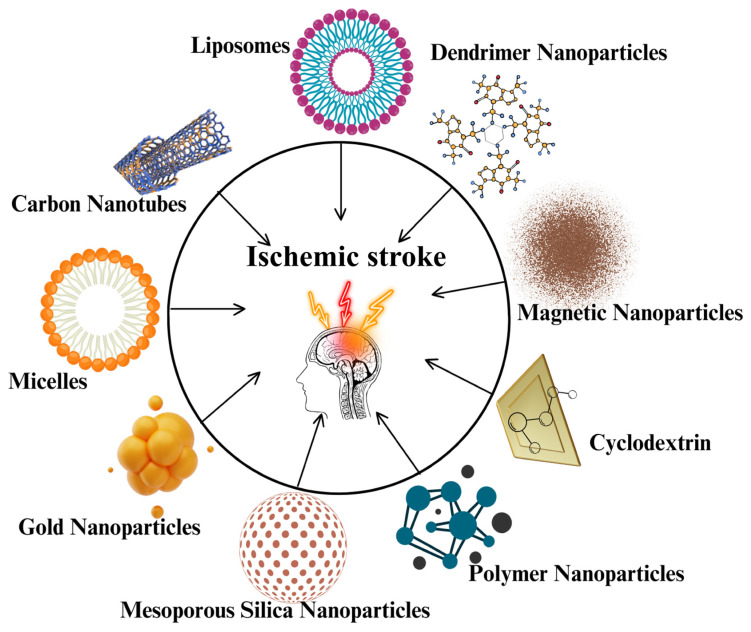
Various types of nanoparticles target ischemic stroke.

The increased detection and alteration of disease-causing processes at the molecular level has made nanotechnology an advanced medical technology [91]. The unique characteristics of nanomaterials (NMs), such as large surface area, are offered to transport drugs or image markers to a specific location within the body [92]. There are various NPs like silica NPs, metallic NPs (gold, silver) [93], carbon nanotubes (CNTs), metal oxide NPs (magnetic NPs), polymeric NPs, dendrimers, liposomes, micelles, etc., are widely used as drug carriers or diagnostic purposes for the management of stroke (shown in Figure 3). Encapsulating drugs or imaging markers protects them from unwanted biological actions. Their capacity to bind to proteins for targeted delivery to specific biochemical areas has enhanced imaging and drug effectiveness in diagnosing and treating strokes. Figure 3 shows the various types of NPs that target ischemic stroke.

Developing theragnostic NPs for stroke diagnosis and treatment involves integrating diagnostic and therapeutic capabilities into a single platform, leveraging NPs’ unique properties. These strategies aim to enhance the precision and efficacy of stroke management by improving drug delivery, targeting, and imaging capabilities. Overcoming the BBB is a major challenge in stroke therapy. NPs can be engineered with surface modifications, such as ligand attachment, to enhance BBB penetration, enabling direct delivery of therapeutic agents to the brain for improved efficacy [91,94]. Moreover, NPs can be designed to target specific pathophysiological processes, such as OS and neuroinflammation, which are critical in IS, thereby enhancing therapeutic outcomes [28].

In diagnostic imaging, NPs are effective contrast agents for modalities like CT, MRI, and fluorescence imaging. These techniques assist in visualizing blood clots and determining their age, which is essential for selecting appropriate treatments [95,96]. Moreover, NPs can function as biosensors to detect pathological markers of stroke, facilitating early diagnosis and real-time monitoring of disease progression [94].

Multifunctional NPs offer theragnostic platforms, such as iodinated NPs (IoNPs), that combine diagnostic and therapeutic capabilities. These platforms enable simultaneous clot visualization and therapeutic intervention, supporting timely and precise treatment decisions [96]. Biomimetic NPs functionalized with cell membranes further enhance targeting and therapeutic precision. By mimicking biological properties, these NPs decrease neurological damage and improve recovery of ischemic brain tissue [97]. Together, these strategies highlight the transformative potential of theragnostic NPs in stroke care.

### 5.1. Nanoparticles and Contrast Agents for Imaging

Nowadays, investigators are trying to obtain high-resolution imaging of the cerebral vasculature, which is becoming increasingly feasible with established approaches modified for use with nanotechnology [98]. One of the most used methods for diagnosing strokes is X-ray-based CT, which separates ischemic from hemorrhagic strokes and identifies areas with varying cerebral perfusion [99]. NP-based contrast agents will significantly enhance the CT and MRI specificity and resolution.

#### 5.1.1. Nanoparticles in MRI Contrast Agents

Conventional MRI contrast agents based on gadolinium have limits on their sensitivity and possible adverse effects. A promising substitute is provided by NPs such as superparamagnetic iron oxide nanoparticles (SPIONs), which are especially well-suited for high-resolution evaluation of the degree of brain injury. The only SPION currently FDA-approved is ferumoxytol, also known as feraheme, which contains iron oxide covered with carboxymethyl dextran. Ferumoxytol’s clinical applications are restricted to iron replacement therapy and imaging in patients with chronic renal disease [100]. Thus, SPIONs offer great contrast, improving the visibility of blood arteries and brain tissue. This improved contrast facilitates a clearer differentiation between hemorrhagic and IS and enables faster identification of ischemic regions. Chitosan/Fe_3_O_4_-encapsulated albumin NPs yield increased signal intensity in the infarcted myocardium, thereby facilitating the detection of analogous pathological features post-stroke [101]. Moreover, polyethylene glycol (PEG)-coated SPIONs with Gadolinium-diethylene triamine penta-acetic acid bring changes in permeability and receptor expression and offer chances for the targeted use of NPs, even though the highly selective BBB generally controls entry to the brain [102].

#### 5.1.2. Targeted Nanoparticles for CT Imaging

AuNPs are emerging as a progressively effective contrast agent in CT imaging. Compared to traditional iodine-based therapies, their elevated atomic number provides superior contrast. Furthermore, these NPs can be functionalized with targeted molecules, such as antibodies or peptides, that specifically bind to biomarkers in areas impacted by ischemia or hemorrhagic stroke [103]. This focused method increases imaging precision and reduces contrast agent dose, minimizing toxicity. The approach reduces contrast agent dose, possibly reducing toxicity, and improves imaging precision. Kim et al. developed GC-contrast AuNPs. CT can show the presence and size of initial and recurrent thrombus by examining NPs [104]. GC-AuNPs’ long circulation half-life allows fibrin matrix anchoring for up to 3 weeks and ongoing monitoring of thrombus formation and thrombolysis. Wang et al. developed polyethylene glycolated BaHoF5 NPs for CTA and CTP imaging to overcome the nephrotoxicity of iodinated contrast agents and improve ischemic stroke detection [105].

#### 5.1.3. Multimodal Imaging

Recently, multimodal imaging has gained tremendous interest in diagnosing brain disorders. Utilizing NPs as hybrid contrast agents in multimodal imaging enables the detection of targets with diverse sensitivity levels, provides comprehensive molecular information, and enhances soft tissue contrast, facilitating accurate quantification. The NPs applied for optoacoustic imaging are mainly carbon-based NPs, AuNPs, bismuth-based NPs, polymer-encapsulated organic NPs, semiconducting polymer NPs (SPNs), conjugated polymers, and novel DNA-based nanocarriers [106,107]. Additional hybrid NPs employed in MRI/optical imaging of cerebral disorders in murine models encompass cobalt NPs, Gd(III)-phthalocyaninate probes, PEGylated polypyrrole NPs conjugated with gadolinium (Gd) chelates, Mn^2+^-doped Prussian blue nanocubes, superparamagnetic iron oxide@Au-labeled stem cells, and copper manganese sulfide nanoplates [108]. Thus, the NPs-based hybrid modality method improves NPs designed for MRI and CT and can provide comprehensive anatomical and functional information about stroke. This dual-modality approach improves the precision of diagnosis and aids in more effective treatment intervention. Table 2 shows recent works exploiting smart diagnostic nano-agents for MR imaging in ischemic stroke.

### 5.2. Biomarker Detection Using Nanosensors

The imaging amenities are prohibitively expensive and, therefore, unavailable in most developing countries. Biomarkers collected from blood, urine, saliva, and CSF aid the diagnosis, prognosis, and therapeutic monitoring of numerous additional diseases where time is less crucial. However, nanosensors, which have a high sensitivity and specificity for diagnosing acute ischemic stroke, are making rapid and accurate biomarker detection possible [6].

#### 5.2.1. Electrochemical Nanosensors

Due to the large SVR, nanosized NPs like AuNPs, CNTs, and graphene at very low concentrations are used to detect stroke biomarkers. These nanosensors detect certain proteins, nucleic acids, or metabolites released during a stroke. An electrochemical signal that can be quantified quantitatively is produced when these biomarkers bind to the sensor surface. This method enables the quick identification of biomarkers for brain damage and stroke, such as neuron-specific enolase, S100 calcium-binding protein B (S100B), and glial fibrillary acidic protein [128]. Other nanosensor technologies are under investigation. These include optical sensing with Raman scattering and nano-optrode detectors, as well as electrochemical detection of the probe–analyte interactions on silver–noocubic structures and platinum NPs [129,130].

#### 5.2.2. Fluorescent Nanosensors

The unique properties of NMs allow fluorescent nanosensors to detect biomarkers and track metabolism in the brain. Fluorescent NPs like quantum dots, CNTs, or dendrimers bind to biomarkers, including oxygen, glucose, nitric oxide, and inflammatory markers, to detect them. These nanosensors emit a fluorescence signal that may be monitored. This sensitive method can detect low-abundance biomarkers in blood or cerebrospinal fluid. Biomarker-based fluorescence probes include metal-ion, protease, and reactive oxygen species-activable probes. The targeted activatable NIR-IIb nanoprobe (V&C/PbS@Ag_2_ Se) in a photothrombotic stroke model detects early ischemic stroke in vivo with good sensitivity [131]. In another investigation, hyaluronic acids (HA) labeled with fluorescein were adsorbed on AuNPs to detect ROS. The ischemia brains had more signals than the normal brains [132]. This technique is favorable for early real-time IS assessment and can be used to make precise diagnoses for other disorders.

#### 5.2.3. Point-of-Care Testing

Several nanosensor platforms are being developed for point-of-care testing, including lab-on-chip devices, NP-based assays, wearable sensors, and portable and smartphone-based devices. By enabling quick and precise stroke diagnosis at the patient’s bedside or in remote locations, these platforms expedite the need for prompt medical attention. For example, lateral flow assays using AuNPs can give a visual readout of biomarker presence in minutes, making them appropriate for emergencies. Such development will enable personalized medicine and targeted treatment. Minimizes unnecessary tests, hospitalizations, and healthcare costs. It also provides accurate and real-time data [133].

Various experiments have been performed using NMs to diagnose and improve strokes [134]. The rapid increase in the utilization of NMs for the treatment of strokes is due to several advantageous features like stability in the blood (i.e., no opsonization by proteins), prolonged time of circulation in the blood, no platelet aggregation, noninflammatory and no activation of neutrophils, avoiding RES, amenable to a small molecule, for instance, peptides, proteins or nucleic acid and controlled release of drugs [135]. The experimental work has shown that NMs mainly focused on the non-invasive imaging of molecularly targeted contrast agents. Various investigations have shown that by using NMs as contrast agents, it is possible to identify and characterize the initial stages of the disease before clinical disorders [136]. The developed NMs (fluorescent, radioactive, superparamagnetic, paramagnetic, electron-dense, and light scattering) were specific in contrast generating for cardiovascular imaging. Among all the NMs, perfluorocarbon (PFC) NPs have been used most widely for strokes due to their long circulation time in the blood, sensitivity, and selectivity against the desired epitope [137]. PFC-NPs are laboratory-synthesized organic compounds in which H atoms are replaced with F atoms. PFC-NPs have a liquid PFC core, such as perfluorooctyl bromide (PFOB), surrounded by a lipid monolayer, which could be functionalized to have various agents for imaging and therapeutic action [138]. PFC-NPs have several unique features, such as prominent contrast-to-noise enhancement, biocompatibility, and biodegradability [139]. All these features can be imaged with MRI or CT. Recently, nanoemulsions have also gained huge importance in the theragnostic of strokes [140]. While using NE for the theragnostic purpose of strokes, the size of NPs is of utmost importance. The size of NPs in NE is the deciding factor of clearance half-life and pharmacokinetic analysis [141]. Experimental studies have shown that a clearance half-life could take between 3 and 6 h, depending on the species. Moreover, researchers further reported that the optical particle size of the NE should be able to penetrate the vasculature and should not be able to be retained by the deep tissues. So, NE of sizes between 200 and 400 nm has gained huge popularity for intravenous applications [142]. Few investigators have shown the benefit of using F in the PFC core for MRI due to their exceptionally sensitive nature for molecular and microenvironmental changes, with essentially no background signal within the body. Some experimental work showed that PFC-NPs could carry a very high paramagnetic payload (6 × 10^4^ to 9 × 10^4^) of gadolinium ions per particle. This has a very high sensitivity for detecting target epitopes with MRI [143]. A notable conclusion has emerged from the collective findings in the investigations above: PFC-NPs exhibit a direct correlation with neovascular density. Using ligand-directed PFC-NPs presents a favorable avenue for non-invasive molecular imaging of angiogenesis. By leveraging the molecular imaging abilities of PFC-NPs, clinicians and researchers may gain valuable perceptions into the processes of angiogenesis, offering a means to evaluate and govern vascular changes associated with diverse cardiovascular conditions [143].

Several investigators have reported improvement in imaging and drug delivery by using cerium oxide NPs [143]. Free radicals are formed due to ischemia associated with strokes, and excessive accumulation leads to ROS [144]. These free radicals are engaged in neural damage. Moreover, this deposition removes electrons from biomolecules, damaging cellular and tissue. CeONPs have been shown to provide a novel nano-pharmacological and neuroprotective approach to diseases associated with OS. Several investigators concluded CeONPs were nontoxic to neuronal (HT22) and macrophage (RAW164) cell lines. In addition, several results show that such CeONPs have antioxidant properties that help in cell survival and decrease the production of free radicals [145].

Several reports have emphasized the utility of PLGA-NPs and other polymeric NPs for theragnostic purposes in the context of strokes [146]. These NPs, including nanospheres, have been explored for their potential to serve both diagnostic and therapeutic functions. However, most of these studies are limited to the model mouse level only.

## 6. Nanotechnology in Stroke Therapy

**Figure 4:**
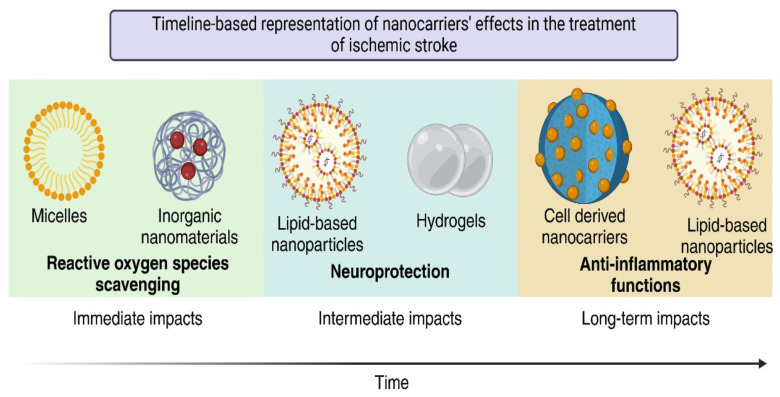
Timeline-based representation of nanocarrier effect in the treatment of ischemic stroke.

The efficacy of potential stroke therapies is greatly diminished by inadequate drug distribution into the blood clot and the central nervous system. Both rt-PA and plasmin raise the possibility of a fatal, symptomatic intracerebral hemorrhage [147,148]. Some practitioners are reluctant to employ thrombolysis due to the danger of intracerebral hemorrhage, which leads to underutilization of treatment [147]. However, nanotechnology helps these chemicals penetrate more easily and be directed toward the desired locations without causing side effects. The application of NPs as nanocarriers for targeted drug delivery in treating ischemic stroke is briefly discussed below. Figure 4 shows the timeline-based representation of the nanocarrier effect in treating ischemic stroke.

### 6.1. Nanoparticle-Based Drug Delivery Systems

In stroke therapy, nanocarriers, including NPs, liposomes, dendrimers, lipid NPs [149], micelles, and polymeric NPs, are modified using various ligands, antibodies, or genes to transport drugs straight to the location of brain damage, increasing effectiveness and reducing adverse effects across the body. They can be specially tailored to target overexpressed receptors in the ischemic or hemorrhagic regions. For instance, Kim et al. loaded tPA on liposomes consisting of egg phosphatidylcholine (EPC), cholesterol (CHOL), sodium cholesterol-3-sulfate (CS), and distearolyphosphatidyl ethanolamine-N-poly(ethylene glycol) 2000 (DSPE-PEG 2000) to enhance the half-life of tPA. Their results revealed that PEGlyted liposomes had extended the half-life of tPA by 21 times compared to free tPA [150]. Another report by Uesugi et al. prepared a zinc-stabilized t-PA-gelatin nano complex that proved an excellent delivery method for t-PA and used local ultrasound radiation to control thrombolytic activity [151]. In another study, rt-PA encapsulated in biocompatible synthetic (polylactic-co-glycolytic acid) coupled with natural polysaccharides (chitosan) had enhanced clot penetration, which led to greater thrombolysis and, ultimately, dose reduction [152]. Besides this, tobacco mosaic virus-mediated iron oxide nanocubes and nanorods conjugated with rtPA had decreased the risk of bleeding in a Rose Bengal model of mouse carotid artery thrombosis as well as enhanced the rtPA targeting and sustained release to vascular thrombi [153]. Magnetic NPs functionalized with various organic or inorganic polymers such as dextran, chitosan, poly[aniline-co-N-(1-one-butyric acid) aniline] are widely used to deliver tPA or other thrombolytic agents to a specific site of thrombosis with minimal dose and side effects [154,155]. Voros et al. used alternating magnetic fields and rtPA-loaded superparamagnetic iron oxide nanocubes to treat thrombi in vitro and in vivo. This strategy led to a restricted rise in temperature to 45 °C, the ideal temperature for thrombolysis, obtaining a 100-fold increase in thrombolysis rates and a ten-fold enhancement of clot disintegration compared to unbound rt-PA [156]. Multifunctional nanocarriers can hold numerous therapeutic drugs. To address several pathological processes related to stroke, functionalized NPs can co-deliver neuroprotective medicines, antioxidants, and anti-inflammatory drugs. This multimodal strategy can improve treatment results by addressing inflammation, OS, and excitotoxicity.

### 6.2. Responsive Nanoparticles

Thrombolytic agent transport can be precisely controlled with stimuli-responsive NPs because they release their payload in response to particular physiological signals like pH, temperature, or enzyme activity [157]. To minimize off-target effects, focused therapy can be enhanced by stimuli-responsive NPs that release drugs in the acidic environment of ischemic brain tissue or reaction to high concentrations of certain enzymes in the stroke-affected area. Principally, pH-responsive and ROS-responsive NPs are among these stimuli-responsive NPs. To treat IS, Cheng et al. created pH-responsive rapamycin-loaded NPs. The encapsulated rapamycin in the NP is carried to the affected brain area, where it is released in the acidic microenvironments of ischemic brain areas, preventing neuroinflammatory reactions and decreasing the amount of the infarct. Another report revealed the induction of cellular autophagy and neuronal apoptosis in IS using an isoliquiritigenin (ISL)-loaded micelle [158]. The rate at which ISL is released from micelle-encapsulated particles falls gradually from pH 7.0 to pH 5.0, and this release is pH-dependent. The ISL release rate reaches its maximum of roughly 60% at pH 7.0 in 48 h and its lowest of roughly 90% at pH 5.0 in 48 h. In addition, drugs are kept at a high concentration in ischemic areas for longer periods because pH-responsive NPs stop them from releasing quickly into circulation. An increased release of ROS in ischemic brain regions permits researchers to develop ROS-responsive nanocarriers for the sustained release of drugs in ischemic stroke [158]. For instance, Zhang et al. designed an upconversion nanoprobe activated by hypochlorous acid (HOCl) to track neuroinflammation in stroke patients [159]. This nanoprobe traversed the BBB via transcytosis, facilitated by a protein linked to the low-density lipoprotein receptor. The difference between inflammation and healthy brain tissue would subsequently be identified by the overproduction of HOCl in ischemic brain regions. Another type of ROS-responsive NPs was developed by Mu et al., where NPs were destructed on identifying by ROS, and ligustrazine enclosed in the NPs would be quickly released to treat IS [160].

### 6.3. Neuroprotective and Neuroregenerative Therapies

In addition to drug delivery, nanotechnology has potential use in neuroprotective and neuroregenerative therapies, which aim to preserve and regenerate ischemic brain tissue. Further extend the half-life and bioavailability of administered medicines, improving their effectiveness. Biodegradable polymeric NPs composed of poly (n-butyl cyanoacrylate) dextran polymers coated with polysorbate 80 (PBCA nanoparticles) are used to deliver neuroprotective agents to specific regions of the ischemic brain [161]. Similarly, liposomes and inorganic NPs like Au, Ag, ZnO, and others are widely used as carriers for neuroprotectant delivery [162]. For instance, short circulation half-lives and low BBB permeability of neuroprotectants like adenosine and brain-derived neurotrophic factor (BDNF) limit their effectiveness. Six times as much BDNF was delivered to the brain and had a longer half-life when mice were given an NP polyion complex (N-BDNF) formulation of BDNF following middle cerebral artery occlusion [163]. This formulation uses diblock copolymers of PEG and poly-Lglutamate to increase CNS delivery. In another study, stroke infarct volume was decreased, and neurological deficit ratings improved when adenosine was added to squalene–adenosine nano complexes, extending the stability of adenosine in plasma from 10 s to 2 h [164]. Reducing OS and inflammation is another key goal of neuroprotective techniques. ROS produced during ischemia-reperfusion injury causes both neuronal damage and an increase in BBB permeability. ROS can be scavenged by antioxidant-loaded NPs, lowering OS and neuronal death. Analogously, anti-inflammatory agents delivering NPs can reduce inflammation while maintaining the integrity of the neuron. For instance, hydrophilic carbon clusters functionalized with poly(ethylene glycol) (PEG-HCCs) are antioxidants that have demonstrated potential in reinstating cerebral blood flow after a traumatic brain injury [165]. Another study has revealed that carbon NPs coupled with antioxidants can prevent animal stroke. PEG-HCCs, a new category of carbon NPs linked to poly(ethylene glycol), exhibited protective effects against OS in a rat stroke model, improving results such as infarct size and neurological function when administered during reperfusion in hyperglycaemic conditions [166]. In an in vitro BBB model, ROS were shown to induce the release of resveratrol, an antioxidant. Additionally, a low-density lipoprotein receptor-binding peptide attached to the NPs facilitated their endothelium targeting by accelerating the degradation of the PLA coating, reducing OS [167].

### 6.4. Nanoparticles for Gene Therapy

NPs that target specific brain cells with nucleic acids like microRNA, RNA, or DNA can aid gene therapy techniques. NPs containing genes encoding for neuroprotective proteins or anti-apoptotic agents can be administered to promote cellular survival and function in the ischemic brain region. This strategy can also silence detrimental genes and contribute to stroke pathophysiology [28]. For example, dendrimers are excellent nanocarriers for gene delivery due to their high transfection efficiency, gene delivery capacity, and low cytotoxicity [168]. Jeon et al. synthesized dexamethasone-conjugated PAMAM G2-Dexa as a carrier for delivering the heme oxygenase-1 gene to cerebral ischemia sites [169]. Their results indicated that the prepared complex reduced inflammation and decreased brain infarct volume. In a related study, Lee et al. enhanced heme oxygenase-1 gene delivery by conjugating PAMAM G2 with histidine and arginine. The findings indicated that modified PG2 demonstrated superior gene delivery efficiency in the brain compared to the unmodified dendrimer [170]. Modified PG2’s low cytotoxicity and high gene delivery efficiency may enhance gene therapy for inflammatory disorders, including ischemic stroke.

### 6.5. Nanoparticle-Based Stem Cell Therapy

Under certain conditions, stem cells (SCs) can multiply, self-renew, and differentiate into various functional cells. Due to these specific features, SCs have demonstrated superior therapeutic efficacy for acute ischemic stroke. Much research has validated the use of NPs to modify SCs to improve the treatment effect for IS. Furthermore, growth factors or genetic material can be delivered to stem cells by NPs, improving their integration and differentiation into injured brain tissue [2]. This method aims to assist in regenerating healthy brain circuits and restoring missing neurons. CXCR4 overexpression on SCs through the SDF-1 α-CXCR4 axis can enhance cell homing efficiency following a stroke. Iron-based NPs such as SPIONs, magnetosome-like 1D ferromagnetic iron oxide nano-chains, and others are involved in improving SC homing in the ischemic brain and require external magnetism to enhance SC absorption [171]. Melanin NPs produced by Tang et al. up-regulate antioxidant defense and prevent apoptosis, which improves mesenchymal stem cells’ (MSCs) therapeutic potential against hypoxic-ischemic injury [172]. Thus, further functional NP research is expected to improve stem cell therapeutic advantages in IS treatment.

### 6.6. Nanomaterials for Scaffold Development

Scaffolds that support structure and encourage the regeneration of brain tissue can be prepared using NMs. Neurotrophic substances or drugs that promote neural repair processes can be released from these scaffolds through engineering. For example, an injectable PEG hydrogel has been developed to break down ROS to improve SC retention and antioxidant protection [173]. Modifying MSCs with palmitic acid-peptide enhanced its populations in the ischemic brain and minimized distribution in adjacent tissues [174]. Such modified MSCs act as potential delivery systems to boost the miR-133b expression in an ischemic lesion and enhance its therapeutic benefits. In another research, lipid-PEG (lipo-PEG)-linked recombinant CXCR4 is applied non-invasively to the surface of MSCs. This surface modification enhanced MSCs’ gradient migration to SDF-1, possibly boosting the therapeutic ability against IS. In addition, by acting as physical barriers and increasing neural stem cell flux, lipid microcapsules decreased the size of infarcts, reduced brain edema, and eventually increased the survival rate of model mice [175]. However, more research is required to improve future stem cell therapy using NMs.

A group of investigators experimented with mice (rodent stroke model) using CeONPs and hypothesized that this nanocomposite has a neuroprotective role. Further, it was found that there was a reduction of about 50% in ischemic cell death by CeONPs [176]. During the investigation, it was revealed that CeONPs led to a 70% reduction in the levels of ischemia-induced 3-nitrotyrosine. Ischemia-induced 3-nitrotyrosine is modified tyrosine residues present in protein and gets induced by peroxynitrite radical. Further, investigators have found CeONPs localized to lipid membranes, mitochondria, and neurofilaments. The investigators suggested this may be of therapeutic relevance as mitochondria, in particular, are known to produce ROS and trigger cell death pathways during ischemia [176].

Some researchers have shown the role of metal oxides NMs like IONPs, especially SPIONs, in improving stroke-related disorders. Here, these SPIONs are mainly used as a contrasting agent in MRI. Research has shown that coating such USPIONs with PEG could inhibit their uptake by RES, and there will be prolonged blood circulation time, which helps in promoting delivery to neural tissues [177]. Moreover, many studies have involved using metallic NPs like Pt, Au, etc., for strokes in the case of mice and *Cenodabrata elegans* in vivo and in vitro. Several investigations have confirmed the possibility of achieving higher spatial resolution and a larger penetration depth in mm compared to MRI and CT. Macrophages are one of the components in the pathophysiology of atherosclerosis, which is a major risk factor for stroke [178]. So, a group of investigators have used AuNPs as a contrasting agent for detecting macrophages in atherosclerotic plaque where the AuNPs were introduced in the macrophages and visualized them ex vivo in atherosclerotic plaques [179,180,181].

In one of the studies, xenon encapsulated in liposomes was ultrasonically guided to brain-stroke treatment after crossing the BBB. Investigators administered Xenon-containing echogenic liposomes in a rat model for up to 5 h after stroke onset. The administered dose was about 7–14 mg kg^−1,^ which was found to minimize the infarct size. Though the human and mouse brains differ significantly, the liposome-based delivery of Xe has shown positive neuroprotection results in patients with acute stroke [182,183,184].

CNTs have shown potential for the treatment of stroke. A group headed by Aldinucci exhibited the immune-modulatory role of multi-walled carbon nanotubes (MWCNTs) with human myeloid dendritic cells, where the investigators suggested the utilization of inert-based-NPs for IS therapy [185]. A group led by Li tried to encapsulate the sulfur hexafluoride (SF6) into platelet membrane (PM) vesicles to formulate biomimetic nanobubbles (PNBs) that integrate theranostics [186].

The various experimental work revealed that 3-n-Butylphthalide (3-n-BPT) could inhibit the aggregation of platelets and minimize the development of a ThB [187]. A team led by Lu tried to elevate the targeted delivery of 3-n-BPT by encapsulating them into Fas ligand antibody conjugated PEG-lipid NPs (FL/NBP/PLNs). Such NPs were found to bind to the microglia in the ischemic regions of the brain. Investigators injected FL/NBP/PLNs intravenously, which was found to penetrate the BBB much more efficiently and specifically deliver 3-n-BPT to the cerebral ischemic regions (CIR) [188].

**Figure 5:**
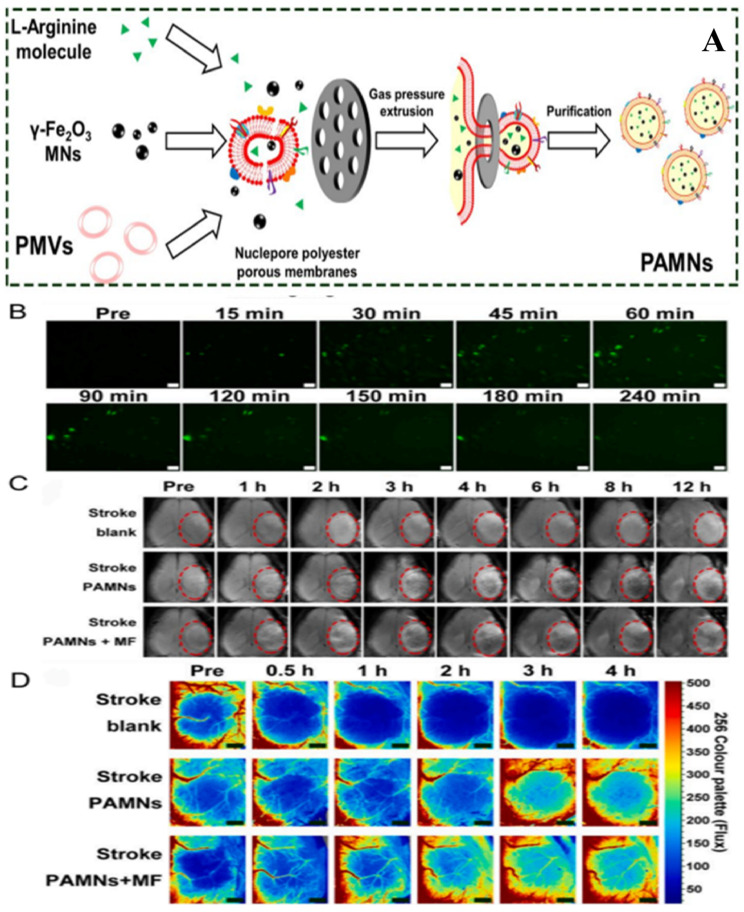
Recanalization therapy of PAMNs for IS: (**A**) Method of preparation of PAMN by extrusion method; (**B**) images of NO generation w.r.t in the bEnd.3 cells stained with DAF-FM DA; (**C**) in vivo T2 MRI before and after saline and PAMNs injection at different time intervals; (**D**) color-coded laser speckle photographs showing blood flow before and after PAMNs injection in ischemic lesion within 4 h. Reprinted (Adapted) with permission from [127]. Copyright 2020 American Chemical Society.

Some of the investigators have used NPs for oxygenation since hypoxia is one major factor in the progress of brain injury. Suppose a surplus amount of O_2_ is supplied to the penumbra in the nascent ischemia phase. In that case, there will be a prolonged thrombolytic time window and an improvement in the viability of neurons. Investigations have shown that hemoglobin is encapsulated into liposomes or PNPs [189]. Table 3 summarizes all the nanomedicines used to effectively treat IS, while Figure 5A–D displays the recanalization therapy of PAMNs for ischemic stroke.

The diverse array of NPs and NM-based therapies showcased in the table reveals the promising potential of nanotechnology in treating stroke. Thrombolysis-based NPs, mainly administered through intravenous (IV) or intra-arterial injection, encapsulate thrombolytic agents like rt-PA or streptokinase, showing enhanced targeting of thrombi, increased drug stability, prolonged half-life, and minimized off-target effects. Similarly, antiplatelet and antithrombosis NPs delivered via IV effectively prevent cerebral ischemia/reperfusion injuries by targeting fibrin-rich thrombi, microglia, or stroke lesions while improving BBB penetration [28,97]. Combination therapies using NPs co-deliver thrombolytic agents and antioxidants or anti-inflammatory drugs prolong drug half-life, enhance BBB penetration, and improve stroke outcomes by targeting multiple stroke-related mechanisms such as OS, neuroinflammation, and neuronal protection. NPs designed for neuroprotection deliver oxygen, scavenge ROS, and modulate inflammatory responses, which reduces brain damage, inhibits neutrophil infiltration, and promotes recovery. Moreover, gene therapy and stem cell-based NPs effectively deliver genes or stem cells to ischemic brain regions, improving recovery, reducing infarct volume, and enhancing neuronal function [264].

While nanotechnology’s potential in stroke management is vast, challenges remain in translating these innovations from the laboratory to clinical practice. Issues such as the scalability of NP production, long-term safety, and regulatory hurdles must be addressed. Additionally, integrating nanotechnology with existing therapeutic modalities could enhance the overall efficacy of stroke treatments. As research progresses, the continued development of nanotechnology-based solutions promises to significantly impact the management of stroke and other complex medical conditions [265].

## 7. Advantages and Disadvantages of the Nanotechnology vs. Existing Approaches

**Figure 6:**
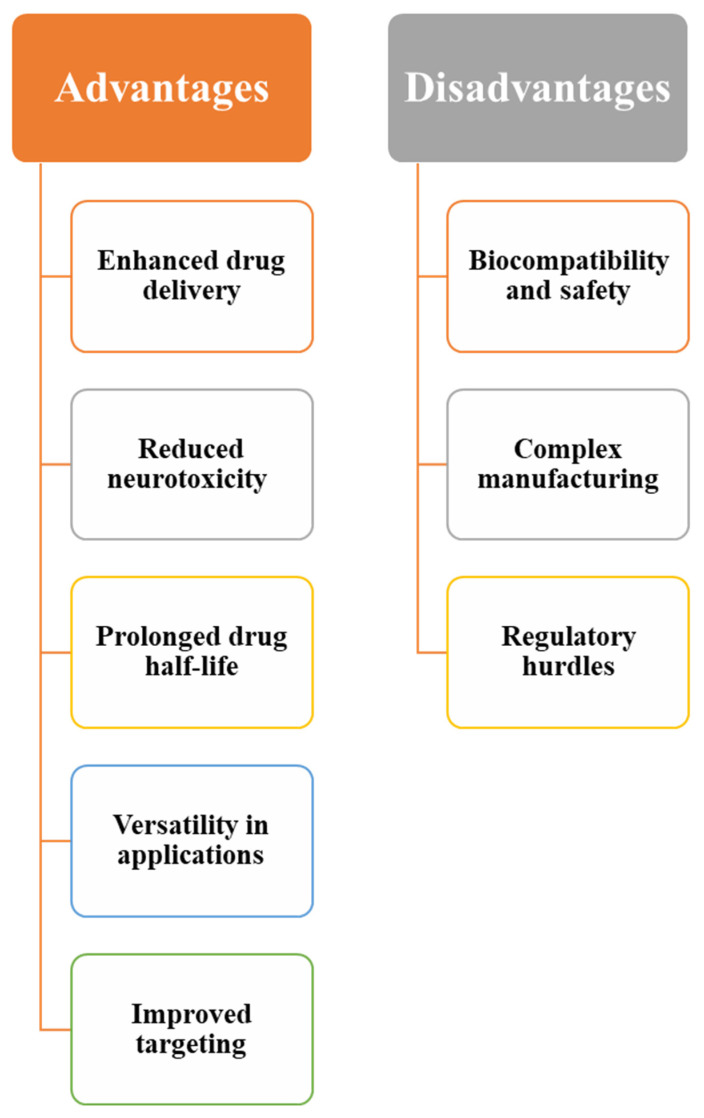
Advantages and disadvantages of nanotechnology for the treatment of strokes.

Nanotechnology offers promising advancements in treating ischemic stroke, presenting advantages and disadvantages compared to standard treatments. Traditional therapies, such as thrombolytic and neuroprotective treatments, face challenges like limited bioavailability, neurotoxicity, and poor BBB penetration [91,134]. Nanotechnology aims to overcome these limitations by enhancing drug delivery and efficacy through NP-based drug delivery systems. However, it also introduces new challenges, such as biocompatibility and safety concerns, which must be addressed for clinical translation [266]. Figure 6 shows the key advantages and disadvantages of the nanotechnology-based stroke treatment.

## 8. Challenges and Future Directions

NPs have advantages and limitations, such as safety and toxicity, which are major concerns among researchers and regulatory agencies. Evidence reveals that some NPs trigger innate immune sensors such as silica dioxide, titanium dioxide, aluminum oxide, and silver [267]. They can trigger inflammasome by interacting with the leucine-rich-containing family, pyrin domain-containing-3, and nucleotide-binding domain, decreasing white cell counts and raising the fraction of neutrophils [268,269]. Additionally, NMs have special characteristics that make it difficult to characterize and standardize them. Due to its complexity, physicochemical characteristics, biocompatibility, and toxicological parameters must be well documented for regulatory bodies such as the FDA and European Medicines Agency [270].

The US FDA has approved nanotechnology-based products for stroke diagnosis and therapy [270]. Despite these advancements, regulatory frameworks are still being improved to address scalability, safety, and efficacy. It is difficult to create regulatory criteria and compare study outcomes across investigations due to the lack of standardized methodology. So, the International Electrotechnical Commission (IEC) and the International Organization for Standardization (ISO) are collaborating to create standards for the creation and evaluation of products based on nanotechnology for the detection and treatment of stroke [28]. To reduce potential toxicity, structural changes of NPs utilizing polyethylene glycol or dextran may be a viable solution to address certain concerns. Furthermore, selecting and assessing the appropriate NPs for an application necessitates caution and thorough safety evaluation. In addition, apprehensions regarding long-term safety and environmental impacts constitute significant ethical and societal dilemmas associated with implementing nanotechnology in medicine. Gaining the public’s trust and acceptance of nanotechnology requires open and honest communication about its hazards and benefits and the inclusion of ethical and public concerns in the regulatory process. Nevertheless, safe production procedures and developing eco-friendly or biodegradable NMs are crucial to reducing environmental influence [271].

## 9. Emerging Trends and Future Prospects in Nanotechnology for Strokes

Despite these challenges, nanotechnology continues advancing with promising developments and prospects for stroke diagnosis and treatment. Personalized nanomedicine, multifunctional NPs, and BBB modification nanotechnology are emerging. Precision advancements in medicine are driving personalized nanomedicine. Tailored nanomedicine customizes nanotherapeutics to patient profiles based on genetic, genomic, and clinical data, enhancing stroke therapy efficacy and safety [28]. This involves developing NPs that target stroke biomarkers or biological pathways. Multifunctional NPs that diagnose and treat (theranostics) draw attention. These NPs administer therapeutic drugs and provide real-time imaging feedback to monitor therapy efficacy and progression of the disease. Incorporating diagnostic and therapeutic activities in one NP provides comprehensive stroke care [272]. The development of NPs that transiently and safely regulate the BBB to improve brain drug delivery is underway. Use NPs to temporarily open the BBB or develop NPs that can cross via receptor-mediated transport or other ways. This method improves brain drug delivery while minimizing systemic exposure. The future of nanotechnology in stroke care includes clinical translation, integration with new technologies, biodegradable and biocompatible NMs, etc. Preclinical research–clinical application integration is a significant future goal. NP-based medicines must undergo extensive clinical trials to prove their safety and efficacy in patients before regulatory approval and clinical use [97]. This project requires collaboration between researchers, physicians, and regulators. Nanotechnology integrated with artificial intelligence and machine learning can potentially enhance stroke detection and treatment methodologies [97]. AI systems can analyze extensive NP-based imaging and biomarker detection datasets to elucidate stroke pathogenesis and inform personalized treatment strategies [273]. Developing biodegradable and biocompatible NMs that can safely decompose and be eliminated from the body is essential. The materials will mitigate long-term toxicity and environmental impacts, enhancing the safety and sustainability of nanotechnology-based stroke therapies.

## 10. Conclusions

Nanoparticles provide improved targeting, increased delivery of drugs, and diagnostic functionalities, particularly in crossing the blood–brain barrier, a major obstacle in stroke therapy. Applying thrombolysis-based nanoparticles, antithrombotic medicines, and combination therapy in managing ischemic strokes demonstrates encouraging outcomes regarding drug efficacy, extended half-life, and diminished off-target effects. Moreover, nanomedicines formulated for neuroprotection, including reactive oxygen species scavengers and anti-inflammatory drugs, have shown efficacy in mitigating brain injury, preventing neutrophil infiltration, and promoting recovery. The amalgamation of gene therapy and stem cell-derived nanoparticles significantly improves recovery results by diminishing infarct size and augmenting neuronal function. Notwithstanding the encouraging outcomes, obstacles persist, especially regarding these nanomedicines’ safety, scalability, and regulatory endorsement. There is an increasing emphasis on developing biodegradable and biocompatible nanomaterials to reduce long-term toxicity and environmental consequences. Future directions encompass the clinical application of these technologies and their incorporation with sophisticated tools such as artificial intelligence for individualized stroke therapy. These advancements position nanotechnology to transform stroke therapy and enhance patient outcomes globally.

## Figures and Tables

**Table 1:** A comparative summary of the different types of pathways through BBB.

Parameters	Passive Diffusion	Adsorption-Mediated Transport	Receptor-Mediated Transport	Carrier-Mediated Transport	Cell-Mediated Transport
Characteristics	Involves the movement of small, lipophilic molecules across the BBB without the need for energy or transport proteins	Involves the non-specific binding of molecules to the cell surface, followed by endocytosis	Utilizes specific receptors on the BBB to transport large molecules, like proteins and peptides, across the barrier	Involves specific transport proteins that facilitate the movement of small molecules, like nutrients and drugs, across the BBB	Involves the transport of cells, such as immune cells, across the BBB
Examples	Small molecule drugs with molecular weight < 400 Da and fewer than 8 hydrogen bonds can cross via passive diffusion	This mechanism is less well-characterized than others and is often considered a minor pathway	Molecular Trojan horse systems exploit receptor-mediated transport to deliver large drugs	CMT transports glucose and amino acids	This pathway primarily involves immune surveillance and response
Limitations/applications/Significance	Most pharmaceuticals do not meet these criteria, limiting their ability to cross the BBB through passive diffusion	Non-specific nature can lead to low efficiency and potential off-target effects	Used in drug delivery systems to enhance the brain uptake of therapeutic agents	Plays a crucial role in supplying essential nutrients to the brain	Complexity of CMT makes it a challenging target for drug delivery

**Table 2:** Recent works exploiting smart diagnostic nano-agents for MR imaging in ischemic stroke.

Material	Design	Effectiveness	In Vivo Model	Technique	References
A low-immunogenic nanoprobe	Nanoprobes developed by coating Fe_3_O_4_ NPs with self-peptides	Nanoprobes effectively tracked stroke progression using SWI and combined DWI tracing	Mouse in a stroke model	MRI	[109]
Catalase-loaded tannic acid NP	Ischemia-homing bioengineered nano-scavenger	Specific clearance of pathogenic elements and inflammation alleviation	Not specified	Not specified	[110]
Cobalt protoporphyrin IX (CoPP) and SPION	Co protoporphyrin-induced nano-self-assembly (CPSP)	Excellent T2-weighted MR imaging and improved MSC survival and neural repair	Middle cerebral artery occlusion mouse model	MRI	[111]
Cu_4.6_O@Dz@PLTM	Cu_4.6_O NP encapsulated by zein-Se-Se-DHA and coated by platelet membrane	Targeting cerebral ischemic lesions, ROS scavenging, neuroprotective effects	Rat model of cerebral I/R injury	MRI	[112]
Fe_3_O_4_ NPs	Low-immunogenic nanoprobe with self-peptide stealth coating	Prolonged blood half-life and enhanced contrast for infarct core, collaterals, and penumbra identification	Not specified	SWI and DWI	[109]
Fluorescent-Magnetite-Nanocluster (FMNC)	Embedding individual magnetite NPs into a polystyrene scaffold coated with two layers of silica and a layer of rhodamine	FMNC-labeled MSCs migrated to and accumulated in the ischemic region after FMNC-labeled MSC transplantation	Mouse in middle cerebral artery occlusion model	MRI	[113]
Iron oxide nanoparticles (IONPs)	P-selectin targeted nanoparticle (MNP-PBP)	Greater T2 effect and early endothelial activation imaging	Transient focal cerebral ischemia in mice	T2 MRI	[114]
IONPs and Au nanorods	Multitheragnostic multi-GNRs crystal-seeded magnetic nanoseaurchin	Enhanced stem cell homing and multimodal imaging	Not specified	Photoacoustic imaging and MRI	[115]
Magnetic nanobubbles (MNBs)	MNBs	MRI and ultrasound imaging surveillance of Neural stem cells (NSCs) based on MNB labeling can be leveraged to provide NSC therapeutic outcomes	Mouse in a stroke model	MRI	[116]
MIRB	MRI or Xenogen imaging combined with the labeling of SPIO-Molday ION Rhodamine-B (MIRB)	MIRB-labeled CD4(+) T cells can be longitudinally visualized in the mouse brain after cerebral ischemia	Mouse in a stroke model	MRI	[117]
MnO_2_ nanozyme	Peptide-templated nanozyme (PNzyme/MnO_2_)	Thrombolytic and neuroprotective actions with prolonged circulation	Mice and rat ischemic stroke models	Not specified	[118]
Neutrophil-camouflaged magnetic nanoprobes (NMNPs)	NMNPs consist of a SPION-loaded PLGA NPs core and a biomimetic neutrophil membrane shell	NMNPs offer a safe and selective option for neuroinflammation imaging	Mouse in middle cerebral artery occlusion model	MRI	[119]
Platelet membrane biomimetic magnetic nanocarrier (PAMNs)	A PAMN loaded with L-arginine and g-Fe_2_O_3_ magnetic NPs (PAMNs)	PAMNs can specifically target different types of damaged blood vasculature	Mouse in a stroke model	MRI	[120]
Polyethylene Glycol (PEG)-ylated Upconversion Nanoprobes (PEG-UCNPs)	High-performance upconversion nanoprobes	High-resolution MRA and MRP imaging with longer circulation time	Acute IS model	MRA and MRP	[121]
Specific Fe_3_O_4_-ArgGly-Asp (RGD	Molecular probes were prepared from an integrin avb3-specific Fe_3_O_4_-Arg-Gly-Asp (RGD)	Fe_3_O_4_-RGD nanoprobes bind to collaterals	Mouse in middle cerebral artery occlusion model	MRI	[122]
SPIONs	Contrast agents were prepared from PEGylated SPIONs	Ability of the PEGylated SPIONs to highlight BBB damage by MRI	Mouse in a stroke model	MRI	[123]
SPIONs	Labeled extracellular vesicles (EVs) from human mesenchymal stem cells (hMSCs)	Enhanced MRI contrast and improved delivery and recovery in ischemic stroke	IS model	MRI	[124]
SPIONs	MRI-visible nanomedicine is developed to co-deliver functional peptides and SPIONs into NSCs	Promotes survival and migration ability of neural stem cells (NSCs). Allows an in vivo tracking of transplanted NSCs with MR	Mouse in a stroke mode	MRI	[125]
VCAM-targeted lipid NPs	Lipid nanocarriers conjugated with antibodies for BBB targeting	62% reduction in cerebral infarct volume with interleukin-10 mRNA	Transient middle cerebral artery occlusion mouse model	MRI	[126]
γ-Fe_2_O_3_ Magnetic NPs	PAMNs	Targeted vascular injury network delineation and pre-protection effect	Acute IS model	T2*-weighted imaging (T2* WI) and DWI	[120]
γ-Fe_2_O_3_ Magnetic NPs	Platelet membrane biomimetic nanocarrier	Rapid targeting and NO generation for vasodilation and reperfusion	Early IS model	Not specified	[127]

**Table 3:** Nanomedicines for effective treatment of IS, their route of administration, and effectiveness.

Mode of Action/Strategies	Route of Administration	Nanoparticle Types/Nature	Effectiveness	References
**Recanalization-based NPs**				
Thrombolysis	Intra-artery injection (IAI)	rt-PA-containing platelet-membrane camouflaged PLGA NPs	Satisfactorily delivered rt-PA to ThB	[190]
	Intravenous injection (IVI)	Porous magnetic (Fe_3_O_4_)-micro rods incorporating rt-PA (tPA-MRs)	Blood clots lysed via rt-PA and the rotating MRs	[191]
		rtPA conjugated to PEG-PCL NPs (rtPA-NP)	Increased rt-PA’s ability to target fibrin and prolonged its half-life	[192]
		Platelet µ particle-inspired nanovesicles (PMINs) for targeting the streptokinase (STK) delivery	Stopped the off-target uptake of STK, actively target the TbH and enzymatically trigger the release of STK	[193]
		pH-sensitive PEG-conjugated urokinase (UrK) nanogels	Extended the release of UrK in the bloodstream and achieved the pH-sensitive release of UrK within the clots	[194]
	NA	PEGylated PLGA NPs containing rt-PA	Increased the thrombolytic activity of tPA	[195]
	NA	RGD peptide conjugated liposomes containing STK	Enhanced targeting of TbH and the release of STK occur immediately following interaction with activated platelets	[196]
	Retro-orbital injection (ROI)	Fucoidan-functionalized polymer NPs loaded with rt-PA	Enhances the efficacy of rt-PA through the targeting of activated platelets in the thrombus	[197]
		Porous soft discoidal PLGA-PEG nanoconstructs containing rt-PA (tPA-DPNs)	Protect rt-PA from degradation in blood circulation and reduce TbH time	[198]
Thrombus-targeting		SPIO-platelet	Reduce neutrophil infiltration, reduce infarct size, and monitor inflammatory neutrophil in real-time	[199]
Antiplatelet	IVI	ThB targeting aspirin polyconjugate NPs	Specifically, targets fibrin-rich ThB and displays strong antithrombotic activity	[200]
		3-n-Butylphthalide was encapsulated within PEG-lipid NPs conjugated with Fas ligand antibodies	Effectively crossed the BBB and selectively attached to microglia in ischemic areas	[188]
		Cilostazol nanodispersions	Effectively prevented cerebral ischemia/reperfusion injury of IS	[201]
Antithrombosis	IVI	Platelet membrane encapsulated with L-arg and maghemite NPs	Efficiently identify the specific stroke lesions and generate nitric oxide in situ utilizing an EMF	[127]
Cavitation	IVI	PNBs are prepared by encapsulating SF6 inert gas into platelet membrane vesicle	Preferentially accumulate at the microvascular compartment of ischemic lesions; facilitate recanalization of the occluded microvascular flow	[186]
**Combination therapy-based NPs**				
TS, Anti-excitotoxicity	IVI	Thrombin-responsive “nanoplatelet” having rtPA and ZL006e	Targets TbH sites; responsively release rt-PA; enhanced BBB penetration potential	[202]
TS, Anti-oxidation	IVI	Self-assembled polyion complex NPs incorporating tissue plasminogen activator (t-PA) and the antioxidant 4-amino-TEMPO	Prolonged half-life of t-PA; elevated antioxidative effect (AOE) of 4-amino-TEMPO	[203]
Scavenging of RONS; Antiinflammation; Inducing protective autophagy of neurons		Microthrombus-targeting and ROS-responsive micelles encapsulating rapamycin	Target the ThB sites; ROS responsively releases rapamycin; combination therapy	[204]
Anti-edema; Anti-oxidation (AO)		Betulinic acid NPs carrying glyburide	Enhanced delivery of glyburide; Achieved the combination of anti-edema and AO effect	[205]
AO		Angiopep-2 and PEG-modified ceria NPs loading edaravone	Enhanced blood circulation time; Enhanced BBB penetration effect; achieved combined ROS elimination effect	[206]
AO	Intracerebral injection (ICI)	Nanocarrier for co-delivery of dexamethasone and heme oxygenase-1 plasmid DNA	Enhanced targeted delivery of dexamethasone and HO-1 plasmid DNA; achieved efficient combinational therapy effect	[207]
Anti-inflammation; AO		Dexamethasone-loaded R3V6 peptide micelles delivering HO-1 plasmid DNA	Exhibited combined anti-inflammatory and antioxidant therapeutic effect	[208]
Anti-inflammatory, antioxidant, and angiogenetic properties	Systemic administration	Various nanotherapies	Reduced infarct size and improved neurological function without significant organ toxicity	[209]
Antioxidant nanomedicines for OS	IVI	Various NPs with antioxidant properties	Reverses neuronal oxidative damage, improves IS outcomes	[210]
Targeting OS and glial overactivation	IVI	Chitosan, thiol ketone, carboxymethyl-β-cyclodextrin, PHSRN peptide	Reduces OS, inhibits astrocyte activation, improves inflammatory microenvironment	[211]
Targeting OS, glutamate excitotoxicity, neuroinflammation, and cell death	IVI	Diverse NPs-based systems	Improved therapeutic outcomes by targeting specific pathophysiological pathways	[29]
ROS-responsive drug release, neuroprotection	IVI	Cu4.6O encapsulated by zein-Se-Se-DHA, coated with platelet membrane	Scavenges ROS, promotes microglia polarization, alleviates cerebral I/R injury	[112]
Antioxidant, anti-inflammatory, mitochondrial targeting		Prussian blue NPs (PB@PDA)	Reduced OS, inflammation, and neuronal apoptosis, improved functional recovery	[212]
ROS-responsive delivery reduced ROS and inflammation	IVI	Chitosan-Bilirubin NPs	Improved motor deficits, decreased infarct volumes, enhanced BBB integrity and neurogenesis	[213]
Targeted delivery, inhibition of NLRP3 inflammasome-mediated pyroptosis		Macrophage membrane-encapsulated	Reduced inflammation, improved therapeutic outcomes in ischemic stroke	[214]
Catalytic and antioxidative activities		CeO_2_	Prolong blood circulation time, reduce clearance rate, improve the penetration ability of BBB, effectively inhibit lipid peroxidation, and reduce neuronal oxidative damage and apoptosis in brain tissue	[215]
**Neuroprotection-based NPs**				
Oxygen supply	IVI	Haemoglobin was encapsulated in Liposome	Enhanced half-life of hemoglobin; efficiently inhibited neutrophil infiltration	[216]
	IVI	PFC emulsion	Significantly reduced brain damage	[217]
Neuroprotection and thrombolysis	Systemic administration	Various nanoplatforms, including liposomes, micelles, and polymeric NPs	Enhanced BBB penetration and reactive oxygen species scavenging	[218]
RONS scavenger	IVI	PEG-modified melanin NPs	Exhibited more potent and safer AOE	[219]
		A novel core-shell NP containing Tempol	Prolonged half-life and reduced effects of Tempol	[219]
		PEGylated ceria NPs	Improved colloidal stability and prolonged circulation time	[219]
		Peptide-modified liposomes loaded with edaravone	Actively targeted surface receptors of leukocytes to migrate into the BBB; delivered edaravone to the damaged neuron cells effectively	[220]
		Ultrasmall carbogenic nanozyme	Exhibited better antioxidative activity than ascorbic acid; behaved effectively for the accidental brain injury treatment	[221]
		Cross-linked polyion complex (PEG-PLL) incorporating SOD1	Accumulated at the damaged cerebral vessels and exert a satisfying AOE	[219]
	NA	PEGylated hydrophilic carbon clusters	Shown AOE similar to superoxide dismutase	[222]
	IVI	Edaravone-encapsulated agonistic micelles	Up-regulated the permeability of BBB; facilitated the specific delivery of edaravone	[223]
	IVI	Pro-Gly-Pro (PGP)-modified PNPs containing catalase	Enhanced delivery of catalase to the ischemic subregions and cerebral neuron cells	[219]
Anti-inflammation	IVI	Platelet-mimetic NPs containing piceatannol and SPIONs	Specifically recognized adherent neutrophils; Enhanced inflammation alleviation effect	[224]
	IVI	Fas ligand antibody conjugated PEG-lipid NPs encapsulated with 3-butyl phthalide	Efficient penetration in the BBB; Remarkably improved brain injury	[188]
	IPI	Liposomal NPs loading acetate	Prolonged blood half-life; reduced GIT irritation of acetate	[225]
	IVI	Neutrophil membrane-derived nanovesicles loading resolvin D2	Specifically adhered to inflamed brain endothelium; efficiently prevented the neural damage	[226]
	IVI	Receptors of (LDLP + neutrophils) co-modified nano-carriers loading scutellarin	Facilitated the penetration of scutellarin through the BBB; significantly improved the neuroprotective effect of scutellarin	[227]
	IVI	Cationic bovine serum albumin-conjugated PEGylated tanshinone IIA NPs	Significantly reduced inflammatory cytokines and decreased the infarct volumes of tMCAO rats	[227]
Anti-inflammation	IPI	PEG/cRGD dual-modified liposomes loading 9- aminoacridine	Reduced cerebral infarct volume; ameliorated the neurological function of tMCAO rats	[228]
Inflammatory-targeting		PLGA	Improved area of injury, greatly improve neurological score and infarct volume	[229]
ROS-scavenging		CeO_2_	Enhance intracerebral uptake while effectively protecting the BBB, greatly reduce harmful side effects and sequelae	[230]
Inflammation suppression via polyprodrug nanomedicine	Intravenous	Neutrophil membrane-camouflaged polyprodrug	Delivers FTY720 effectively, reduces cardiotoxicity, reprograms microglia	[231]
**RONS scavenging + Anti-inflammation**				
RONS scavenging + Antiinflammation	IVI	4T1 cancer cell-inspired nano vehicle loaded with succinobucol	Preferentially targeted the IS lesions and effectively penetrated the BBB	[219]
	IVI	mPEG-b-PLA NPs encapsulating curcumin	Reduce OS; protected the BBB; inhibited M1 microglial activation	[219]
	IVI	CeO_2_@ZIF-8 NPs	Enhanced catalytic and AOE; improved stroke therapeutic effect	[232]
	IVI	c-(RGDyK) peptide-functionalized exosomes loading curcumin	Recognized the integrin αvβ3 on the reactive cerebral vascular endothelial cells and preferentially accumulate at the CIR	[219]
	Intrathecal injection	Molybdenum-based polyoxometalate nanoclusters	Bypass the BBB and quickly reach the CIR	[233]
	Lateral cerebral ventricle injection	Amine-modified SWCNTs	Efficiently protect against neuron damage	[234]
	IVI	Curcumin liposomes functionalized with MSC membrane	Efficient penetration in the BBB and accumulation in the ischemic regions; enhanced survival rate of MCAO mice	[235]
Free radical scavenger and oxygen regulator	IVI	Mn_3_O_4_ was encapsulated into T7 peptides attached to natural erythrocyte	Efficient penetration through the BBB; protected the neurocytes before and after thrombolysis	[219]
**Gene therapy (GT)**				
	IVI	Cationic lipid-assisted PEG-PLA NPs encapsulated with C3-siRNA (NPsiC_3_)	Decreased C3 expression in microglia; Reduced microglial neurotoxicity	[236]
	IVI	Self-assembled NPs (HSAP-NP/pHO1) composed of hypoxia-specific anti-RAGE peptide (HSAP) and HO1 plasmid (pHO1)	Increased gene delivery and expression in the ischemic tissues	[237]
	IVI	M2 microglia-derived exosomes	Promoted neuronal function recovery by inhibiting neuronal inflammation	[238]
	ICI	HO1-mRNA was delivered with DA-PEI2k to form into HO1-mRNA/DA-PEI2k complex	Exhibited higher gene expression; significantly reduced the infarct volume	[239]
	Intranasal administration	High mobility group box 1 (HMGB1) siRNA delivered by polyamidoamine (PAMAM)s dendrimer of e-PAM-R	Efficiently realize the knockdown of HMGB1; Markedly suppress the infarct volume of MCAO rats	[240]
	Carotid injection	RGD peptides cationic polymer interact with HIF-1α plasmid DNA	Effectively targeted vascular endothelial cells; Significantly improved recovery of tMCAO rats	[241]
SiRNA delivery and imaging		SPIO	Infarct volume was reduced, functional defect and partial anisotropy (FA) values were increased, and fiber count was increased	[242]
Endothelial progenitor cell therapy and GT	Intracardiac injection	EPCs transfected with superparamagnetic iron oxide/siPHD2 complexes	Enhanced expression of CXCR4 and HIF-1α; elevated homing and survival ability of EPCs	[243]
**Neuronal stem cell therapy and GT**				
Neuronal stem cell therapy and GT		NSCs transfected with ROS-responsive B-PDEA/BDNF plasmids polyplexes	Enhanced BDNF expression in the mouse brain; Enhanced the functional recovery of the ischemic brain	[244]
		NSCs transfected with SPIONs and siRNA targeting NgR gene (siNgR)	Exhibited in vivo imaging of transfected NSCs; Silence the NgR gene; Enhanced the differentiation of NSCs	[245]
MSCs and GT	IVI	MSCs transfected with MFIONs-based gene complexes	Produced high gene transfer efficiency in MSCs; Improved the homing of MSCs in the CIR; Significant reduction in the mortality of ischemic mice and recovered the ischemic brain function	[246]
	ICI	MSCs transfected with core-shell NPs (CPMSN@125I-SD)	Realize real-time PA imaging of MSCs in the mouse brain and SPECT imaging of the ischemic mouse brain; Maintain superior therapeutic effect for IS	[247]
Enhanced BBB penetration	IVI	Polymeric nanocarriers	Improved neuronal protection, increased therapeutic effects, extended half-life, targeted delivery	[248]
Lipid nanocarriers for enhanced drug delivery	IVI	Lipid NPs	Improves pharmacokinetic and pharmacodynamic properties, enhances BBB permeation	[26]
Targeted delivery to inflamed BBB	IVI	Lipid nanocarriers conjugated with antibodies	Reduces cerebral infarct volume, enhances drug concentration in ischemic brain	[126]
Surface-modified ligands for enhanced BBB penetration	IVI	NPs with surface modifications	Improved drug permeability and targeting efficiency	[91]
Stimuli-responsive changes for controlled drug release	Systemic	Stimuli-responsive nanomedicines	Enhanced drug delivery and reduced side effects	[249]
Targeting vascular cellular adhesion molecules at the BBB	IVI	Lipid NPs	Significant reduction in cerebral infarct volume	[126]
Cell membrane-functionalized for active targeting	IVI	Biomimetic NPs	Improved targeting and reduced neurological damage	[97]
Cell membrane-derived nanovehicles (CMNVs) to targeted delivery, controlled release	IVI	Erythrocyte, thrombocyte, neutrophil, macrophage, neural stem cell, and cancer CMNVs	Enhanced targeting and reduced drug dosage, minimized side effects	[250]
Dual-modified extracellular vesicles (RA-EVs) for enhanced BBB penetration and ischemic region targeting	IVI	Adipose-derived stem cell extracellular vesicles modified with RGD and Angiopep-2 peptides	Decreased infarct volume, apoptosis, and neurobehavioral deficits	[251]
	IVI	Lipid NPs (B-rLNPs) incorporating brain-derived lipids	Reduced infarct volume, neurological deficits, BBB leakage, selective homing to cerebral ischemic areas	[252]
Lipid NPs for Olig2 mRNA delivery	IVI	CD140a-targeted lipid NPs loaded with Olig2 mRNA	Enhanced remyelination and cognitive function, promote remyelination and improve cognitive deficits	[253]
N-acetyl cysteine amide (NACA) and CeO_2_ NPs for antioxidant delivery to reduce OS	IVI	CeO_2_ NPs with NACA	Reduced OS and improved antioxidant capacity	[254]
Cell-penetrating peptide for lysosomal degradation of SIRT5 to alleviate neuroinflammation	IVI	Membrane-permeable peptide targeting SIRT5	Reduced brain infarct area and improved neurological functions	[255]
pH-sensitive		mPEG-b-P (DPA-co- HEMA)-Ce6	Drug concentration in ischemic sites, neuroprotective effects	[256]
Targeted-AMD3100, pH-sensitive release	IVI	Multifunctional polymeric NPs (PTT NPs, or ASPTT NPs)	Target delivery to ischemic brain tissue with high efficiency, accelerate drug preferentially release to ischemic brain tissue	[257]
ROS/pH-sensitive release	IVI	Boronic ester-modified dextran polymeric NPs	Prolong systemic circulation of NR2B9C, enhanced active targeting effect of NR2B9C on ischemic area, and alleviated ischemic brain injury	[258]
PH-sensitive release	Intravenous	MnO_2_	Reduce OS, promote transformation ofM1-type microglia to M2-type microglia, enhance the survival of damaged neurons	[259]
Protease-responsive		PEG, PCL, enzyme cleavable peptides	High efficiency in penetrating ischemic brain area, safe material and low toxicity	[260]
Lesions-targeting		IONPs	Promote the anti-inflammatory response, angiogenesis, and anti-apoptosis of ischemic brain injury, reduce volume of cerebral infarction, improve motor function	[261]
BBB-targeting, antioxidative, anti-inflammatory, antiapoptotic	IVI	Metal–Organic Framework (MOF)	Enhanced drug retention, controlled release, reduced cerebral infarction, improved nerve function	[262]
Combination of exosomes and macrophage membranes for targeting		Satellite NPs (MEps)	Mitigated ischemic-reperfusion injury, enhanced neurogenesis, strong biocompatibility	[263]

## Data Availability

The data can be availed by requesting the corresponding author.
